# Documentation of smoking in scheduled asthma contacts in primary health care: a 12-year follow-up study

**DOI:** 10.1038/s41533-022-00309-4

**Published:** 2022-10-21

**Authors:** Jaana Takala, Iida Vähätalo, Leena E. Tuomisto, Onni Niemelä, Pinja Ilmarinen, Hannu Kankaanranta

**Affiliations:** 1 Department of Internal Medicine, Seinäjoki Central Hospital, Seinäjoki, Finland; 2 Department of Respiratory Medicine, Seinäjoki Central Hospital, Seinäjoki, Finland; 3 Tampere University Respiratory Research Group, Faculty of Medicine and Health Technology, Tampere University, Tampere, Finland; 4 Department of Laboratory Medicine, Seinäjoki Central Hospital, Seinäjoki, Finland; 5 Tampere University, Tampere, Finland; 6 Krefting Research Center, Department of Internal Medicine and Clinical Nutrition, Institute of Medicine, University of Gothenburg, Gothenburg, Sweden

**Keywords:** Asthma, Clinical trial design, Population screening

## Abstract

Smoking among asthmatics is common and associates with poorer asthma control, more rapid lung function decline and higher health care costs in dose-dependent manner. No previous real-life studies exist, however, on how smoking status and pack-years are documented in scheduled asthma contacts in primary health care (PHC) during long-term follow-up, and how often patients are advised to quit smoking. In this real-life 12-year follow-up study, we showed that out of all scheduled PHC asthma contacts (*n* = 603) smoking was mentioned only in 17.2% and pack-years only in 6.5%. Smoking data was not recorded even once in 70.9% of never smokers, 64.7% of ex-smokers and 27.3% of current smokers. Smoking including pack-years were mentioned more often if nurse took part on the scheduled contact. For current smokers, smoking cessation was recommended only in 21.7% of their scheduled contacts. Current smokers used more antibiotics and had more unscheduled health care contacts during follow-up.

## Introduction

Worldwide, asthma is a common heterogenic disease consisting of multiple different phenotypes^1–3^. Adult-onset asthma is often associated with lifestyle or environmental factors such as smoking and obesity^1,3^. These factors may contribute to suboptimal asthma control alongside allergy, rhinitis, gastroesophageal reflux, comorbidities, problems in inhalation technique, and poor adherence to asthma medication^1,2,4,5^. Smoking is known to associate with reduced effectiveness of inhaled steroids^6^, poorer asthma control^5,7–9^, rapid decline in lung function^4,10^, and higher health care costs^3^. Patients with asthma may become vulnerable to the adverse effects of smoking on lung function already before asthma is diagnosed^10^. The number of smoked pack-years correlate with frequent hospitalizations, higher number of comorbidities, symptoms and asthma severity in a dose-dependent manner^9,11^. Among patients with adult-onset asthma smoking history of ≥10 pack-years is associated with accelerated lung function decline independently of whether the patient has stopped smoking or not^4^. Patients with smoking history of ≥10 pack-years often present with poorly controlled asthma^7,9^. These results not only highlight the importance of interventions aimed at smoking cessation in early phase among patients with adult-onset asthma but also underscore the importance of routine screening and careful assessment of lifelong smoking history during follow-up.

Smoking poses an enormous threat to public health worldwide, killing more than eight million people every year although the prevalence of smoking has been declining at global level by 23% over the past 12 years^12^. Smoking among asthma patients varies between countries approximately from 10 to 26%^13,14^, and it is usually equally common in general adult population^15^. Patients with respiratory disease have a greater and more urgent need to stop smoking^16^ due to the obvious benefits of smoking cessation for the decreased prevalence of chronic obstructive pulmonary disease (COPD)^17^ and better symptom control in asthma^15^. Unfortunately only modest smoking cessation rates have been reported in asthma^18,19^. Asking about smoking and encouraging smoking cessation varies greatly between general practitioners (GP) in different countries in Europe and U.S., and is often not implemented as recommended^20,21^.

According to current guidelines, smoking status and history should be evaluated in asthma^1,22^ and in COPD^23^ and recorded systematically in medical records^23^. It can be argued that if patients’ smoking status or a discussion about smoking cessation was not documented during the planned patient contacts, it was not done. To the best of our knowledge no previous long-term real-life studies exists on how smoking status including pack-years are documented in real-life scheduled asthma contacts in primary health care (PHC). Thus, the main aim of this study was to assess how smoking and pack-years were documented during scheduled asthma contacts in PHC and if differences exist between contacts with GP, nurse, or both. The second aim was to evaluate how often patients were advised in smoking cessation and to assess how smoking status affected their asthma control and health care use.

## Methods

### Study design and population

The present study was a part of Seinäjoki Adult Asthma Study (SAAS), which is a single-center (Department of Respiratory Medicine, Seinäjoki Central Hospital, Seinäjoki, Finland) 12-year real-life follow-up study of 203 patients with new-onset asthma diagnosed at adult age (≥15 years). The details of the SAAS study protocol with inclusion, exclusion and specific diagnostic criteria have been published previously (eTable 1)^24^. More than 94% of the patients diagnosed with novel asthma in the study site were recruited to the SAAS study^24^. Diagnosis of new-onset asthma was made by a respiratory physician based on typical symptoms and was confirmed by objective lung function measurements^24,25^. Smokers and patients with concomitant COPD or other comorbidities were not excluded^24^. After the diagnosis was confirmed and the medication started the patients were treated and monitored by their personal physicians mostly in PHC according to the Finnish National Asthma Programme^24–26^. After 12 years (mean 12.2, range 10.8–13.9 years) a total of 203 patients completed a follow-up visit in respiratory department in secondary health care where asthma status, disease control, comorbidities and medication were evaluated using structured questionnaires and lung function was measured^24^. The participants of the follow-up visit gave written informed consent to the study protocol approved by the Ethics committee of Tampere University Hospital, Tampere, Finland. In addition to the data gathered at diagnostic and follow-up visits, all data of asthma-related health care contacts during 12-year period was collected from PHC, occupational health care, hospital, and private clinics as previously prescribed^24,25^. The flowchart of the SAAS study is shown in Supplementary Fig. 1. The SAAS study is registered at www.ClinicalTrials.gov with identifier number NCT02733016.

**Fig. 1:**
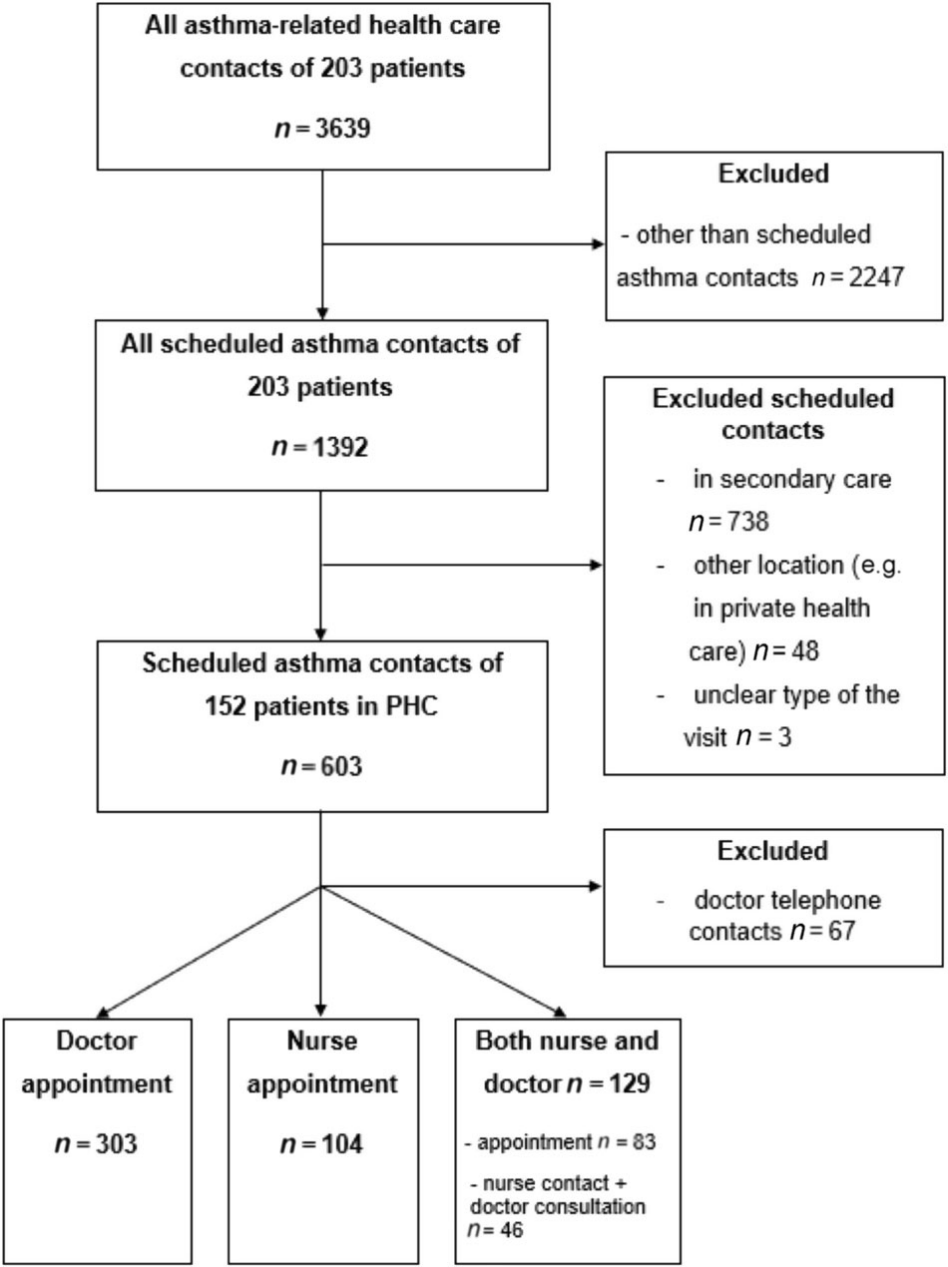
The flowchart of the study. The distribution of scheduled asthma contacts.

In the present study, all asthma-related health care contacts (*n* = 3639) of the 203 patients during the 12-year follow-up period were assessed (Fig. 1). Of those, we included scheduled PHC asthma follow-up contacts of 152 patients, the total number of scheduled contacts in PHC being 603 (Fig. 1). The excluded 51 patients did not have any scheduled asthma contacts in PHC^25^. In this study, we considered both scheduled follow-up contacts in health care centers and in occupational health care as PHC follow-up contacts. Out of the 603 scheduled asthma contacts, 303 were doctor appointments, 104 nurse appointments, 129 were contacts when both nurse and GP were involved in the asthma follow-up visit and 67 consisted of planned GP telephone contacts (Fig. 1). The occurrence of not only these PHC contacts (*n* = 603) but also the overall participation of the 203 patients in scheduled asthma contacts during SAAS study period are described in our previous studies^25,27^ as well as the definition of Finnish PHC and the organization of asthma management in the health care centers^25^.

### Assessment of smoking

Smoking status was determined at the diagnostic visit and at the 12-year follow-up visit in secondary health care. The patients were categorized to never smokers, ex-smokers, or current smokers according to their current and past smoking behavior. Those who reported having never smoked regularly were considered never smokers. Those who had smoked regularly but had quitted smoking before the clinical visit were considered ex-smokers. Those who smoked currently were classified as current smokers. Lifelong cumulative exposure to tobacco was evaluated by assessing smoked pack-years (20 cigarettes per day for 1 year). All documented smoking data collected at scheduled asthma contacts during 12-year follow-up period in PHC were evaluated and analyzed.

### Lung function, inflammatory parameters, computation of adherence, and other clinical measurements

Lung function measurements were performed with a spirometer according to international recommendations^28^. The annual FEV_1_ decline was calculated by measuring the change between the highest FEV_1_ measurement available during the first 2.5 years after the diagnosis and start of inhaled corticosteroid (ICS) therapy (Max_0–2.5_) and FEV_1_ at the follow-up, and by dividing the sum with elapsed time. Fraction of exhaled nitric oxide (FeNO) was measured with a portable rapid-response chemiluminescent analyzer according to American Thoracic Society standards^29^ (flow rate 50 mL s^−1^; NIOX System, Aerocrine, Solna, Sweden). Venous blood was collected, and white blood cell differential counts were determined. Total immunoglobulin E (IgE) levels were measured by using ImmunoCAP (Thermo Scientific, Uppsala, Sweden). Laboratory assays were performed in an accredited laboratory (SFS-EN ISO/IEC 17025:2005 and ISO 15189:2007) of Seinäjoki Central Hospital. Patients completed Airways Questionnaire 20 (AQ20)^30^, Asthma Control Test (ACT)^31^ and COPD Assessment Test (CAT)^32^. Assessment of asthma control was performed according to the Global Initiative for Asthma (GINA) 2010 report^33^. Classification of asthma therapy steps was assessed by daily medication regimen according to the Global Initiative for Asthma (GINA) 2019 guideline [Step 1 and 2: >0–400 μg ICS as budesonide equivalents OR daily LTRA OR low-dose ICS-formoterol; Step 3: >400 μg ICS as budesonide equivalents OR low-dose ICS + LABA OR low-dose ICS + LTRA; Step 4: >800 μg ICS as budesonide equivalents OR medium dose ICS and at least one second controller (LABA, LAMA, LTRA, xanthine, chromones); Step 5: >800 μg ICS as budesonide equivalents and at least one second controller (LABA, LAMA, LTRA, xanthine, chromones) OR biologics]^34^.Assessment of severe asthma was performed according to the ERS/ATS severe asthma guideline 2014^35^.

Adherence to ICS medication was evaluated by comparing the dispensed doses to the prescribed doses for the whole 12-year period as described in our previous studies^36,37^. The prescribed dose in each patient was calculated based on medical records, and the dispensed ICS, short-acting β_2_-agonist (SABA) and oral corticosteroids were obtained from the Finnish Social Insurance Institution, which records all purchased medication from all Finnish pharmacies^36,37^. The 12-year adherence and annual adherence for each patient was calculated by using specific formulas as previously described taking into account aspects from Medication possession ratio (MPR) and proportion of days covered (PDC)^36^. SABA usage was determined by counting all dispensed SABA canisters during 12-year follow-up together and dividing the sum by 150 puffs [SABA canisters (150 puff/canister) during 12 years].

Information on alcohol consumption was assessed by detailed structured questionnaires. Heavy alcohol consumption was evaluated by self-report, GT-CDT index or by both. Assessment of alcohol consumption was performed according to the US definitions for alcohol consumption by portions/week (portion indicates 14 g alcohol)^38^. Serum levels for carbohydrate-deficient transferrin (CDT) were measured by a turbidimetric immunoassay (TIA) after ion exchange chromatography (%CDT, Axis-Shield, Oslo, Norway) and plasma γ-glutamyltransferase (GT) concentration was measured using enzymatic colorimetric assay, as standardized against IFCC (International Federation of Clinical Chemistry and Laboratory Medicine). More detailed information on GT and CDT measurements and on calculating the GT-CDT index have been previously reported^39^.

### Statistical analysis

Continuous data is expressed as mean (SD) for variables with normal distribution and for parameters with skewed distributions, medians and 25–75 percentiles are shown. The Shapiro–Wilk test was used to assess normality. Two group comparisons were performed by using Student’s t test for continuous variables with normal distribution, Mann–Whitney test for continuous variables with skewed distribution and Pearson Chi-square test or Fisher’s exact test for categorized variables. Two-sided p-values were used. A p value <0.05 was regarded as statistically significant. Statistical analyses were performed using the SPSS software, version 27.0.1.0 (IBM SPSS, Armonk, NY).

### Reporting summary

Further information on research design is available in the Nature Research Reporting Summary linked to this article.

## Results

### Characteristics of the study population

Out of the total number of 203 patients in SAAS study population, 152 participated in scheduled asthma follow-up visits in PHC. In total, these patients had 603 scheduled asthma contacts in PHC, thus, each patient had approximately four planned contacts during the 12-year follow-up period as described previously^25^. Most of the patients with scheduled PHC asthma follow-up contacts were women. At follow-up visit, mean age was 59 years and every second patient had a history of smoking. Approximately one-third of the patients had uncontrolled asthma according to GINA 2010^33^. The main characteristics of the study population (*n* = 152) at follow-up visit are shown in Table 1.

**Table 1:** Characteristics of the patients (*n* = 152) with scheduled follow-up contacts in primary health care at 12-year follow-up visit.

	Patients with scheduled asthma follow-up contacts in primary health care
Number of patients	152
Female *n* (%)	96 (63.2)
Age (years)	59 (13)
BMI (kg/m^2^)	28.5 (5.9)
Smokers (ex or current) *n* (%)	76 (50.0)
Atopic *n* (%)^a^	51 (37.2)
Rhinitis *n* (%)	109 (71.7)
Uncontrolled asthma *n* (%)^b^	46 (30.3)
Daily ICS in use *n* (%)	125 (82.2)
Daily SABA in use *n* (%)	21 (13.8)
Daily LABA in use *n* (%)	78 (51.3)
Daily add-on drug in use *n* (%)	85 (55.9)
≥1 oral corticosteroid course during 12-year follow-up *n* (%)	50 (33.6)
Pre-BD FEV_1_ (%)	87 (17)
Post-BD FEV_1_ (%)	91 (17)
Pre-BD FEV_1_/FVC	0.74 (0.67–0.79)
Post-BD FEV_1_/FVC	0.76 (0.70–0.80)
FeNO (ppb)	11 (5–19)
Blood eosinophils (×10^9^/l)	0.15 (0.10–0.27)
Total IgE (kU/l)	61 (23–154)
Co-existing COPD (post FEV_1_/FVC < 0.7 and pack-year ≥10) *n* (%)	19 (12.6)
ACT score	21 (19–24)

If not otherwise mentioned, shown are mean (SD) or median (25th–75th percentiles). Add-on drug = long-acting β_2_-agonist, leukotriene receptor antagonist, theophylline, and/or tiotropium in daily use.

*BMI* Body Mass Index, *ICS* inhaled corticosteroid, *SABA* short-acting β2-agonist, *LABA* long-acting β2-agonist, *BD* bronchodilator, *FEV*_*1*_ forced expiratory volume in 1 s, *FVC* forced vital capacity, *FeNO* fraction of nitric oxide in exhaled air, *ACT* asthma control test.

^a^At least one positive skin prick test of common allergens.

^b^Assessment of asthma control was performed according to the Global Initiative for Asthma (GINA) 2010 report.

### Changes in smoking habits during the 12-year follow-up

**Fig. 2:**
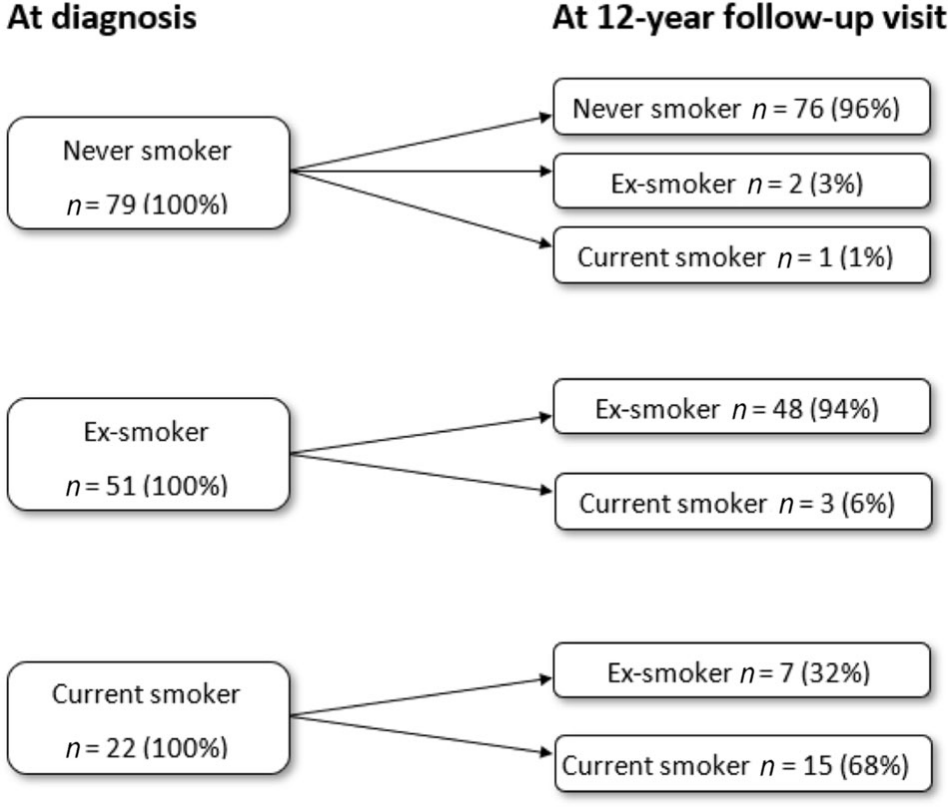
Smoking status changes. Smoking habit changes during the 12-year follow-up.

The patients having scheduled contacts in PHC were divided into three groups according to their smoking status at the study baseline (never smoker, ex-smoker, and current smoker). Out of 152 patients, 52.0% (*n* = 79) were never smokers, 33.5% (*n* = 51) were ex-smokers and 14.5% (*n* = 22) were current smokers at the time of the asthma diagnosis (Fig. 2). Out of the 79 patients representing never smokers, 96% could still be classified as never smokers at the 12-year follow-up visit. Among ex-smokers 6% had changed their status into active smokers. After the diagnosis, 32% of smokers had managed to quit smoking (Fig. 2). In this study population, active smoking reduced from the 14.5 to 12.5% during the 12-year follow-up after asthma diagnosis.

### Recording of smoking data in scheduled asthma contacts

**Fig. 3:**
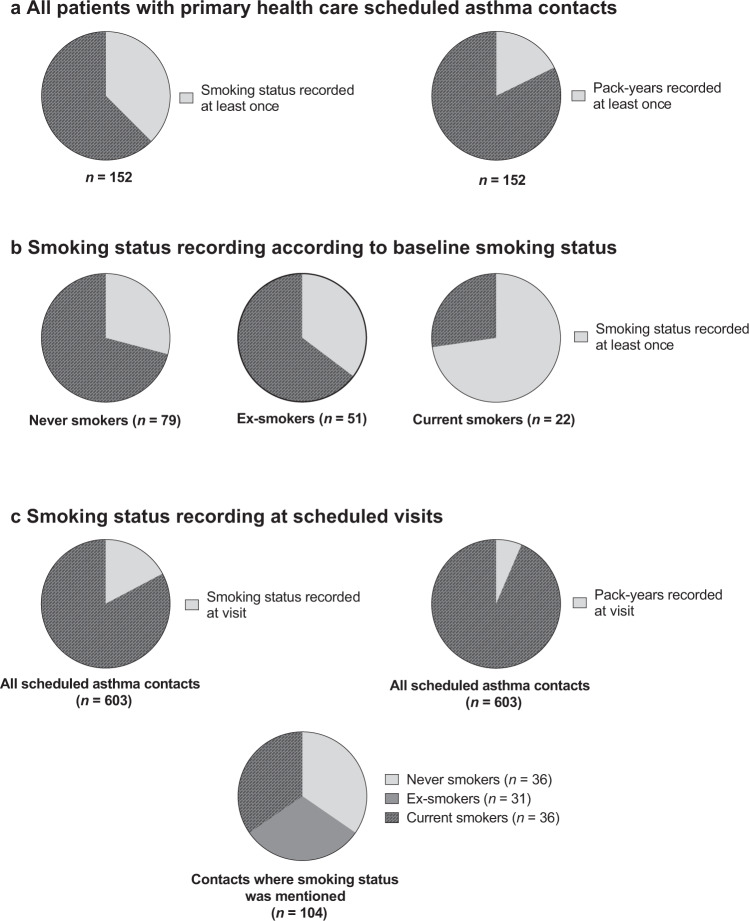
Distributions of smoking data recordings in scheduled asthma contacts. Distributions of smoking data recordings according to **a** study population, **b** baseline smoking status, and **c** number of scheduled contacts.

To evaluate the assessment of smoking in the scheduled asthma contacts (*n* = 603), all documented smoking-related data were collected and analyzed from the follow-up period. Out of the 152 patients having scheduled contacts in PHC, smoking status was not reported even once for 95 patients (62.5%) and smoked pack-years were not calculated even once for 125 patients (82.2%) (Fig. 3a). Smoking status was not recorded even once in 56 (70.9%) never smokers, in 33 (64.7%) ex-smokers and in 6 (27.3%) current smokers (Fig. 3b). Out of all 603 scheduled asthma contacts, smoking status was recorded only in 104 contacts (17.2%) and pack-years were calculated in 39 contacts (6.5%) (Fig. 3c). In most visits where pack-years had been mentioned (34 contacts, 5.6%), it was stated that patient was never smoker (i.e., 0 pack-years) and in only 5 visits (0.8%) pack-years were evaluated in a current or ex-smoker. Of the 104 contacts in which smoking status was recorded, 36 visits were done by never smokers, 32 visits by ex-smokers and 36 visits by current smokers (Fig. 3c).

**Fig. 4:**
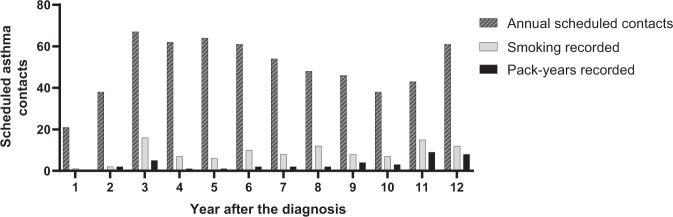
Recording of smoking data during annual scheduled asthma contacts. Smoking data recording in all scheduled asthma contacts (*n* = 603) in primary health care during 12-year follow-up among 152 patients with adult-onset asthma.

The occurrence of the scheduled asthma contacts (*n* = 603) of this study population (*n* = 152) during 12-year follow-up in PHC is described more precisely in our previous study^25^. During the years 1–12 after diagnosis, the annual number of scheduled contacts among 152 patients varied from 21 to 67^25^. At the same time recording of smoking status varied annually from 4.8 to 34.9% (on average 16.8%) being the weakest during the first two years (Fig. 4). The annual average of calculation of pack-years was 6.5%.

### Documentation of smoking data in patients with ex-smoking or current smoking history

**Fig. 5:**
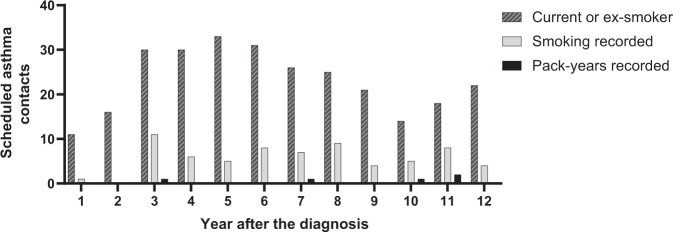
Recording of smoking data during annual scheduled asthma contacts among ex- or current smokers. Recording of smoking status and pack-years in scheduled asthma contacts over a period of 12 years in patients being either current or ex-smoker.

In the assessment of asthma, the knowledge on the smoking status can be considered highly important especially if the patient is ex-smoker or current smoker^40^. Out of all 603 scheduled contacts, 45.9% (*n* = 277) were contacts in which the patient was either current or ex-smoker. Among these patients (*n* = 73), smoking was not mentioned even once with 39 patients (53.4%) and pack-years were not calculated even once with 68 patients (93.2%). During these contacts, smoking was recorded on average in 23.9% and pack-years in 2.1% of the annual contacts (Fig. 5).

### Recording of smoking habits and smoking cessation advising among current smokers

**Fig. 6:**
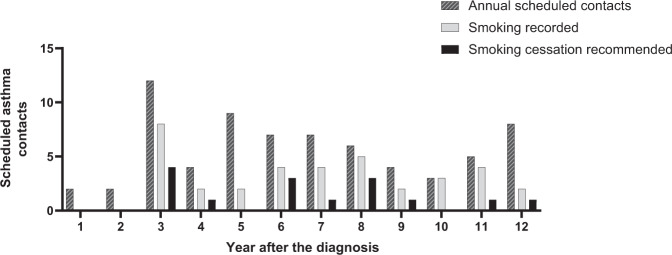
Smoking data recording and smoking cessation advising during 12-year follow-up for patients being current smokers at the study baseline. The total number of all scheduled asthma contacts of current smokers (*n* = 22) in primary health care was 69.

To explore how smoking habits were screened and if smoking cessation was recommended for current smokers in PHC, we analyzed the 69 scheduled asthma contacts of the patients who were current smokers (*n* = 22) at the study baseline (Fig. 6). The annual number of scheduled asthma contacts among current smokers varied between 2 and 12 during the follow-up period. During these contacts, smoking was recorded on average in 49.3% of annual contacts. Pack-years were poorly recorded, and number of currently smoked cigarettes was more often mentioned than pack-years (35.4 vs. 6.3%) (eFig. 2). Smoking cessation was rarely recommended, a total of 15 times during 12-year period corresponding to 21.7% of visits in which the patient was an active smoker. Out of all current smokers, 13 (59%) had not had smoking cessation advise during scheduled asthma follow-up contacts. As shown in Fig. 2, 32% of smokers (*n* = 7) managed to quit smoking during the follow-up, and out of these 43% (*n* = 3) had received smoking cessation advise during scheduled contacts.

### Documentation of smoking data according to the health care professional

To evaluate if differences exist in the recording of smoking according to who is responsible for the patient in the office-based asthma follow-up contact, we divided the follow-up contacts (*n* = 603) in PHC into three groups (Fig. 1). Out of all planned follow-up contacts, 303 were GP contacts, 104 were asthma-nurse contacts, and in 83 contacts patient met first nurse and GP thereafter. In 46 contacts, nurse met patient and then consulted GP, and these contacts were included to the last group (total number of combined GP and nurse contacts *n* = 129). We excluded 67 follow-up contacts related to planned GP telephone contacts only (Fig. 1). Smoking status was mentioned in 13.5% of doctor contacts, in 27.9% of nurse contacts and in 25.6% of contacts when both nurse and GP took part in the contact. Pack-years were mentioned only in 2.4% of scheduled contacts when patient met only GP. Smoking and pack-years were mentioned more often if nurse participated in the scheduled contact (Table 2).

**Table 2:** Recording of smoking in scheduled primary health care office-based visits (*n* = 536) according to the health care professional encountering the patient.

	Doctor contact (*n* = 303)	Nurse contact (*n* = 104)	Both doctor and nurse contact (*n* = 129)	*p-*value
Smoking status mentioned *n* (%)	41 (13.5)	29 (27.9)	33 (25.6)	<0.001
Pack-years mentioned *n* (%)				
No	296 (97.7)	90 (86.5)	111 (86.0)	<0.001
Yes	2 (0.7)	1 (1.0)	2 (1.6)	
Mentioned, that non-smoker	5 (1.7)	13 (12.5)	16 (12.4)	

### Characteristics of the patient groups according to the study baseline smoking history

The above results show that smoking status and pack-year history were infrequently recorded in scheduled asthma follow-up contacts. To evaluate the importance of smoking status to the outcome of asthma, we divided the patients (*n* = 152) into two groups according to smoking status at the study baseline: 79 patients were never smokers, and 73 patients were ex-smokers or current smokers. At the 12-year follow-up, most of the patients having positive smoking status were men (54.8%) and had median 17.0 (6.3–29.3) pack-years smoking history. They had more partially and uncontrolled asthma, had lower FEV_1_ and FEV_1_/FVC ratio, steeper annual decline in lung function, and more symptoms according to CODP Assessment test (CAT)^32^ when 26.0% of them had also co-existing COPD (Table 3). Never smokers had more allergy and asthma medications in use. Every fourth ex-smoker or current smoker (25.0%) were also heavy users of alcohol. No significant differences were found in health care use or in comorbidities (eTable 2).

**Table 3:** The characteristics of the study groups according to the baseline smoking status at 12-year follow-up visit.

	Never smoker *n* = 79	Ex-smoker or current smoker *n* = 73	*p-*value
Male n (%)	16 (20.3)	40 (54.8)	**<0.001**
Age (years)	58.8 (13.9)	60.2 (12.3)	0.486
BMI (kg/m^2^)	27.8 (4.4)	29.2 (7.1)	0.123
Smoking status mentioned *n* (%)	23 (29.1)	34 (46.6)	**0.030**
Mentioned ≥2 times	7 (8.9)	14 (19.2)	0.061
Pack-years mentioned *n* (%)	22 (27.8)	5 (6.8)	**<0.001**
Pack-years of smokers	–	17.0 (6.3–29.3)	**–**
Asthma control GINA 2010^a^ *n* (%)			
Well controlled	39 (49.4)	15 (20.5)	
Partially controlled	19 (24.1)	33 (45.2)	<**0.001**
Uncontrolled	21 (26.6)	25 (34.2)	
ACT score	22 (19–24)	21 (19–23)	0.549
CAT score	10 (5–17)	13 (8–19)	**0.041**
Average daily prescribed ICS dose among 12 years (µg budesonide equivalents)	800 (507–934)	841 (696–1054)	**0.019**
Average daily dispensed ICS dose among 12 years (µg budesonide equivalents)	474 (319–788)	712 (386–898)	0.098
Total adherence in ICS medication during 12 years (%)	78.5 (46.4–100.5)	81.5 (47.4–93.7)	0.720
Add-on drug in daily use *n* (%)	44 (55.7)	41 (56.2)	>0.999
SABA puffs/week	1.6 (0.97–3.67)	2.4 (0.99–4.47)	0.247
Number of asthma or/and allergy medication in use	3 (2–3)	2 (2–3)	**0.046**
Pre-BD FEV_1_ (%)	91.9 (15.2)	82.4 (17.7)	**<0.001**
Post-BD FEV_1_/FVC	0.78 (0.71–0.82)	0.73 (0.68–0.79)	**0.004**
Annual change in lung function from Max_0–2‚5_ to follow-up^b^			
FEV_1_ (ml/year)	−32.6 (−54.2–19.7)	−52.5 (−66.2–25.9)	**0.005**
FEV_1_ %/year	−0.26 (−0.80–0.39)	−0.70 (−1.18–0.09)	**0.004**
Co-existing COPD (Post FEV_1_/FVC < 0.7 and pack-year ≥10) *n* (%)	0	19 (26.4)	**<0.001**
Heavy alcohol consumption (evaluated by self-reports, GT-CDT index or by both) *n* (%)^c^	9 (11.4)	18 (25.0)	**0.035**

If not otherwise mentioned shown are mean (SD) or median (25th–75th percentiles). Bold values indicates statistically significant p-values. Add-on drug = long-acting β_2_-agonist, leukotriene receptor antagonist, theophylline, and/or tiotropium in daily use.

*BMI* Body Mass Index, *ACT* asthma control test, *CAT* COPD assessment test, *ICS* inhaled corticosteroid, *SABA* short-acting β_2_-agonist, *BD* bronchodilator, *FEV*_*1*_ forced expiratory volume in 1 s, *FVC* forced vital capacity, *GT-CDT* gammaglutamyltransferase-carbohydrate-deficient transferrin-index.

^a^Assessment of asthma control was performed according to the Global Initiative for Asthma (GINA) 2010 report.

^b^Annual change in FEV_1_ during 12 years of follow-up (ΔFEV_1_ from point of maximal lung function within 2.5 years after start of therapy to the 12-year follow-up visit).

^c^Assessment of alcohol consumption was performed according to the US definitions for alcohol consumption by portions/week. For men, heavy drinking is defined as consuming 14 portions or more per week. For women, heavy drinking is defined as consuming 7 portions or more per week. Portion indicates 14 g alcohol.

### Characteristics of the ex-smokers and current smokers at 12-year follow-up visit

We subsequently explored how ex-smoking or current smoking affected the disease characteristics at the end of the follow-up. For this purpose, we divided the patients into two groups according to smoking status at 12-year follow-up visit: ex-smokers (*n* = 57) and current smokers (*n* = 19). At the end of the follow-up period, most of the current smokers were women, they were younger [mean age 53.2 (10.1)] and had a median of 22.2 pack-years (from 15.6 to 33.5) smoking history. Current smokers had more unscheduled contacts in health care and used more antibiotic courses during the follow-up. Out of all current smokers (*n* = 19), smoking status had been recorded at least once with 14 patients (73.7%) but more often only with 8 patients (42.1%) during the 12-year follow-up. Almost half of current smokers (47.7%) were heavy users of alcohol and none of them had education over 12 years (Table 4). Current smokers had lower fraction of NO in exhaled air (FeNO) and 26.3% of them had also thyroid disease, but no significant differences were found in other comorbidities, asthma control, asthma severity, lung function, or other laboratory parameters (Table 4 and eTable 3).

**Table 4:** Characteristics of ex-smokers and current smokers at the 12-year follow-up visit.

	Ex-smoker *n* = 57	Current smoker *n* = 19	*p-*value
Male *n* (%)	35 (61.4)	6 (31.6)	**0.034**
Age (years)	61.8 (12.6)	53.2 (10.1)	**0.008**
BMI (kg/m^2^)	29.1 (6.8)	29.0 (7.7)	0.987
Pack-years of smokers	12.8 (3.5–24.0)	22.2 (15.6–33.5)	**0.011**
Heavy alcohol consumption (evaluated by self-reports, GT-CDT index or by both) *n* (%)^a^	9 (16.1)	9 (47.4)	**0.011**
In working life *n* (%)	21 (36.8)	12 (63.2)	0.062
Length of education ≥12 years *n* (%)	7 (12.3)	0	**0.004**
Smoking status mentioned *n* (%)	21 (36.8)	14 (73.7)	**0.008**
≥2 during 12-year follow-up	6 (10.5)	8 (42.1)	**0.003**
Pack-years mentioned during 12-year follow-up *n* (%)	2 (3.5)	3 (15.8)	0.096
FeNO (ppb)	12.0 (7.0–23.0)	5 (2.5–8.0)	**<0.001**
Uncontrolled asthma *n* (%)^b^	22 (38.6)	5 (26.3)	0.145
Severe asthma *n* (%)^c^	3 (5.3)	3 (15.8)	0.161
ACT score	21 (19–24)	21 (19–22)	0.266
CAT score	13 (7–18)	14 (9–19)	0.580
Average daily dispensed ICS dose among 12 years (µg budesonide equivalents)	609 (331–838)	770 (490–958)	0.231
Total adherence in ICS medication during 12 years (%)	76.7 (46.7–93.2)	85.7 (41.1–98.3)	0.686
Purchased antibiotic courses during the follow-up *n* (%)	8 (2–13)	12 (5–19)	**0.040**
≥2 OCS course for asthma during 2 years before follow-up *n* (%)	13 (22.8)	1 (5.6)	0.165
Purchased OCS courses during the follow-up (mg/year)	80 (0–188)	92 (0–217)	0.990
≥1 hospitalization due to any respiratory related reason *n* (%)	13 (22.8)	6 (31.6)	0.543
Unscheduled contacts	4 (1–10)	9 (3–17)	**0.012**
Co-existing COPD (post FEV_1_/FVC < 0.7 and pack-year ≥10) *n* (%)	15 (26.8)	4 (21.1)	0.765
Thyroid disease *n* (%)	3 (5.3)	5 (26.3)	**0.020**

If not otherwise mentioned, shown are mean (SD) or median (25th–75th percentiles). Bold values indicates statistically significant p-values.

*BMI* Body Mass Index, *GT-CDT* gamma*glutamyltransferase*-carbohydrate-deficient transferrin-index, *FeNO* fraction of NO in exhaled air, *ACT* asthma control test, *CAT* COPD assessment test, *ICS* inhaled corticosteroid, *OCS* oral corticosteroid, *FEV*_*1*_ forced expiratory volume in 1 s, *FVC* forced vital capacity.

^a^Assessment of alcohol consumption was performed according to the US definitions for alcohol consumption by portions/week. For men, heavy drinking is defined as consuming 14 portions or more per week. For women, heavy drinking is defined as consuming 7 portions or more per week. Portion indicates 14 g alcohol.

^b^Assessment of asthma control was performed according to the Global Initiative for Asthma (GINA) 2010 report.

^c^Assessment of severe asthma was performed according to the ERS/ATS severe asthma guideline 2014.

## Discussion

In this real-life 12-year follow-up study, we showed that smoking was infrequently addressed in PHC in a regionally representative sample of asthma patients in Finland. Out of all 603 scheduled asthma contacts in PHC, smoking status was mentioned only in 17.2% and pack-years in 6.5% of contacts. Out of the total of 152 patients having visits in PHC, smoking status was not reported even once for 62.5% of the patients and smoked pack-years were not calculated even once for 82.2%. Smoking data were not recorded even once in 70.9% of never smokers, 64.7% of ex-smokers, and 27.3% of current smokers. We found that smoking and pack-years were mentioned more often if nurse took part on the scheduled contact. Among the population representing current smokers at baseline, 32% succeeded to quit smoking during the 12-year follow-up. For current smokers, smoking cessation was recommended only approximately in every fifth (21.7%) follow-up visit, and 59% of these patients had not received smoking cessation advise during scheduled asthma contacts. As expected, patients with smoking history showed poorer outcomes in asthma.

One of the main goals of the Finnish National Asthma Programme was reduction in respiratory irritants, such as smoking and environmental smoking^26^. Previously, it has been shown that smoking reduced from 24% to 18% among asthmatics in Finland during 2001–2010^41^. In our study, half of the asthma patients in PHC were ex-smokers or current smokers. In this study population, active smoking declined from the 14.5% to 12.5% during the follow-up. In 2018, 15% of working aged men and 13.0% of women were daily smokers in Finland^42^. Thus our study population did not differ significantly from general population or from typical population with asthma^42,43^.

To the best of our knowledge, no previous real-life studies exist on how smoking status and the quantities of pack-years are documented in scheduled asthma contacts in PHC in long-term follow-up, and how often during the follow-up the patients are advised to quit smoking. Studies assessing documentation of smoking often include, also, patients with COPD or other chronic diseases^13,21,44–47^. A previous review reported that failure to adequately document smoking history is common in asthma but also in other conditions^44^. On the other hand, a single study from U.S. focusing on treatment recommendations in asthma has indicated high percentages of smoking-related reports in patient records^48^. In our study, out of the total of 152 patients, smoking status was assessed and documented only in 37.5% of adult asthmatics. Among ex-smokers and current smokers (*n* = 73), smoking was documented in less than half (47%) and pack-years less than in 7% of the patients. Recent registry-based study from Finnish secondary care showed that among asthmatics smoking status was documented in 61% of patients and that clinicians documented smoking more actively in years 2016–2018 than in years 2010–2012^47^. The patients included in the previous study were either diagnosed for the first time with disease or they were referred to secondary care for treatment optimization^47^. Thus, it could be argued that due to this fact smoking was more likely to be documented and, on the other hand, use of preliminary information forms is more common in secondary care in Finland, which may have made smoking information more visible. During SAAS-study, general background information forms, which would contain, e.g., smoking and pack-year information, were not in use in PHC in the study region. Our results, suggesting that smoking was recorded in less than every fifth scheduled asthma contact and pack-years in <7% of contacts, may reflect the possible national neglecting attitudes toward smoking habits in PHC in accordance with previous study showing that smoking habits was mentioned only in 42% of asthma referral letters sent to respiratory department^49^. In addition, in more recent Finnish study, recording of smoking status was visible in 14.2% of the PHC referrals to operative care and very little attention was paid to the need for preoperative smoking cessation in PHC^50^. During the Finnish National COPD program written information on smoking habits in records increased from 16.6% of all patients with respiratory symptoms in 1997 to 53.2% in 2002 and in COPD group from 45.0 to 84.3%^51^. However, duration and amount of smoking were also poorly documented^51^. Based on our results, overall amount of current tobacco use was more often mentioned than pack-years among smoking asthmatics.

Many of the studies are conducted from the perspective of what has been done by the GP, but less is known whether differences exist according to the professional that meets the patient (GP, nurse of both). Swedish study showed that documentation of smoking habits was more frequently carried out in asthma nurse consultations compared to GP contacts (78 vs. 28%)^45^. Our results are similar, but although smoking data was recorded more often when asthma nurse participated in the scheduled contact, still smoking was mentioned only in <70% and pack-years in <15% of these contacts.

According to current guidelines patients with asthma should strongly be encouraged to quit smoking^1^^,^^22^. Cessation support and treatment should be provided in all health care settings and by all health care providers^12^. Study based on self-reports showed that 41% of the patients with lung conditions reported receiving information from doctor or nurse about the health effects of smoking both before and after their diagnosis, while 13% reported that they had received guidance only before diagnosis, 31% after diagnosis and 15% of patients not at all^46^. It is suggested that even if smoking is screened it is less likely that smoking patients are advised to quit^52^. In addition, diagnosis of respiratory disease does not seem to motivate people to quit smoking^53^. In our study smoking cessation was rarely recommended to asthmatics, a total of 15 times during 12-year period corresponding to 21.7% of visits in which the patient was an active smoker. Out of all current smokers, 32% succeeded to quit smoking but at the same time over half of the patients did not receive cessation advice, and few of the non-smokers and ex-smokers began to smoke. The proportion of patients who received guidance to quit smoking (41%) was in line with found by Stridman et al. (38%)^13^. In our study, out of the patients who succeeded to quit smoking, 43% had received smoking cessation advise during scheduled asthma contacts in PHC. Recent Finnish study showed that smoking cessation was discussed with 55.4% of current smoker asthmatics in secondary care, but still these patients were seldom referred to nurse-managed smoking cessation program^47^.

Our results raise doubts whether PHC professionals are truly aware of the importance of evaluation of smoking and smoked pack-years among asthma patients, and whether these are better screened with COPD patients as smoking is a well-known risk factor for the disease^23^. Recent study from Finland showed that smoking status was documented more frequently in COPD and sleep apnea patients in secondary care, and that smoking cessation was discussed more frequently in COPD (59.5%) and type I diabetes (61.0%) than in asthma (55.4%)^47^. In U.S. was also found that PHC physicians provided counseling more frequently to smokers with COPD than smokers without chronic diseases (46% vs 25%) or with asthma (31%)^21^. In Sweden, smoking cessation support was offered to 27% of 12–17 year old adolescent asthmatics, to 38% of adult-asthmatics and to 51% of the patients with COPD^13^. Based on our study and previous studies^49,50,54,55^ it could be argued that smoking cessation activities in PHC in Finland have remained inadequate despite asthma guidelines^1,22^ and national smoking cessation guidelines since 2002^56^. Although there is strong evidence for the benefits of quitting smoking, its implementation is shown to be poor not only in respiratory diseases but also in many other conditions^21,44,53,57^. A previous study from U.S. suggested that among smokers with chronic smoking-sensitive diseases, 50–72% of the patients received no counseling about smoking cessation^21^. Study from Finland reported inadequate smoking cessation counseling when number of smokers who had been advised by at least one health care professional during the preceding year varied from 24% to 26% for men and 22% to 26% for women^58^. In more recent study Hirvonen et. al. showed that in Finnish secondary care encouragement to smoking cessation varied between seven common chronic disease from 41% to 61%^47^. Several factors may influence in physicians’ engagement in smoking cessation including physician-related, patient-related, and health care organization-related factors^20,52,59^. Among pregnant asthmatics smoking may be better screened and the benefits of smoking cessation more thoroughly advised^60^. It is also suggested that smoking cessation counseling is more frequently provided to young patients and, overall, if more time is available for the contact^21^. The probability for quitting smoking is shown to be more likely with higher levels of education and fewer years of smoking^53^.

Our results further showed that patients with ex-smoking or current smoking history had poorer outcome in asthma. In line with previous studies^7,9^ they had more symptoms, poorer lung function and more partially controlled and uncontrolled asthma. One quarter of them had also co-existing COPD. In our study almost every second current smoker was also heavy alcohol drinker. It could be argued that this may increase the risk that smoking is not actively addressed while heavy alcohol consumption is shown to associate with poorer participation in asthma follow-up^27^. Alcohol and smoking may also create adverse synergistic interactions on lung function^61^. At the end of the study period, out of all current smokers almost 70% were female, they were younger, had lower education level, more unscheduled health care contacts and used more antibiotics. There were no differences between ex-smokers and current smokers in lung function, in hospitalizations or in use of oral corticosteroids. Current smokers had lower FeNO and more thyroid disease which both have shown to associate with smoking^62,63^. The above results indicate that among smoking asthmatics the assessment of smoking and pack-year history and active advising of smoking cessation is crucial and should be done more actively in every health care contact.

Our study has several strengths. The diagnosis of asthma was made by a respiratory physician and the diagnosis was based on typical symptoms and objective lung function measurements showing reversibility of airway obstruction. The study population well represents a typical PHC population with asthma when smokers or patients with co-morbidities were not excluded^24,43^. In this study all scheduled asthma contacts in PHC were evaluated including both nurse and GP contacts. Thus, documentation of smoking habits could be accurately assessed. Overall, in this real-life study we had 603 scheduled contacts, which may be expected to yield a representative sample of real-life adult asthma population. Possible weakness of our study is that our results may not represent entire Finland. It is, however, more likely that similar neglecting attitudes towards smoking habits in asthma care are also prevailing throughout the world. Differences may occur, for example, in the use of structured preliminary information forms, that could make tobacco use status more identifiable for clinicians. Possible limitation is also that smoking habits may have been screened and smoking cessation advised but it has not been documented to the patient records. On the other hand, according to the good clinical practice, measures taken shall be recorded in medical records or otherwise it can be interpreted that it has not been carried out. It should also be noted that the number of current smokers at the follow-up (*n* = 19) was low, which might lead to loss of power in statistical analyses.

Based on our results and the known facts of dose-dependent harms of smoking to asthmatics, it appears that PHC practitioners should pay increasing attention to the evaluation of smoking habits among asthmatics, including quantitative estimates of the number of pack-years. This should be done already at the time of asthma diagnosis and followed during each subsequent asthma contact. The importance of assessment and recording of smoking and pack-years as well as smoking cessation should be increasingly emphasized in asthma treatment guidelines. In addition to smoking, the possible use of snuff and e-tobacco should be screened, as they have also been suggested to impair lung health^64,65^.The use of ready-made phrase templates could help to collect smoking data more efficiently during asthma follow-up contacts. According to recent national tobacco statistics decline in the number of smoking adults seems to have stopped in Finland^42^. The PHC has the main responsibility in counseling and managing smoking cessation. The first component of brief patient counseling for tobacco cessation starts with asking about the smoking status^12,56^. Based on our findings, smoking cessation should be provided more actively to asthmatics by ensuring adequate resource, guidance, support, and time for this work. Group counseling would provide the opportunity for peer support to the patients and enable effective use of health care resources but also virtual support systems for smoking cessation should be developed and effectively utilized. The presence of an electronic medical record reminder has suggested to be valuable tool in efforts to promote smoking cessation^66^ and its use should be further assessed in existing patient information systems. Further studies are needed to evaluate how other essential factors affecting asthma control are considered in scheduled asthma contacts in PHC.

As a conclusion, in this real-life 12-year follow-up study we showed that smoking and pack-years were poorly addressed in PHC in Finland. Out of all scheduled asthma contacts (*n* = 603), smoking status was recorded only in 17.2% and pack-years only in 6.5%. Smoking data were not recorded even once to 70.9% of never smokers, to 64.7% of ex-smokers, and to 27.3% of current smokers. Smoking and pack-years were documented more often if nurse took part on the scheduled contact. Smoking cessation was rarely recommended. In the future, it is essential that PHC practitioners pay more attention to evaluation of smoking habits and the number of pack-years among asthmatics.

## Supplementary information

Reporting Summary

Supplementary Material

## Data Availability

All data generated or analyzed during this study are included in this published article and its Supplementary Information File. According to ethical permission and patient data-protection laws of Finland, single patient data cannot be made available.
