# Supplementary material for: Documentation of smoking in scheduled asthma contacts in primary health care: a 12-year follow-up study
Source: NPJ Prim Care Respir Med. 2022 Oct 21;32:44. doi: 10.1038/s41533-022-00309-4 (PMC9587006; doi:10.1038/s41533-022-00309-4)
Supplement: Supplementary file 1 — Reporting Summary [file 41533_2022_309_MOESM1_ESM.pdf]

nature portfolio

Corresponding author(s): Jaana Takala

Last updated by author(s): Oct 4, 2022

Reporting Summary

Nature Portfolio wishes to improve the reproducibility of the work that we publish. This form provides structure for consistency and transparency in reporting. For further information on Nature Portfolio policies, see our Editorial Policies and the Editorial Policy Checklist.

Statistics

For all statistical analyses, confirm that the following items are present in the figure legend, table legend, main text, or Methods section.

n/a	Confirmed	
☐	☒	The exact sample size (n) for each experimental group/condition, given as a discrete number and unit of measurement
☐	☒	A statement on whether measurements were taken from distinct samples or whether the same sample was measured repeatedly
☐	☒	The statistical test(s) used AND whether they are one- or two-sided<br>*Only common tests should be described solely by name; describe more complex techniques in the Methods section.*
☒	☐	A description of all covariates tested
☐	☒	A description of any assumptions or corrections, such as tests of normality and adjustment for multiple comparisons
☐	☒	A full description of the statistical parameters including central tendency (e.g. means) or other basic estimates (e.g. regression coefficient) AND variation (e.g. standard deviation) or associated estimates of uncertainty (e.g. confidence intervals)
☐	☒	For null hypothesis testing, the test statistic (e.g. F, t, r) with confidence intervals, effect sizes, degrees of freedom and P value noted<br>*Give P values as exact values whenever suitable.*
☒	☐	For Bayesian analysis, information on the choice of priors and Markov chain Monte Carlo settings
☒	☐	For hierarchical and complex designs, identification of the appropriate level for tests and full reporting of outcomes
☒	☐	Estimates of effect sizes (e.g. Cohen's d, Pearson's r), indicating how they were calculated

Our web collection on statistics for biologists contains articles on many of the points above.

Software and code

Policy information about availability of computer code

Data collection	Microsoft Excel.
Data analysis	Statistical analyses were performed using SPSS software, version 27 (IBM SPSS, Armonk, NY).

For manuscripts utilizing custom algorithms or software that are central to the research but not yet described in published literature, software must be made available to editors and reviewers. We strongly encourage code deposition in a community repository (e.g. GitHub). See the Nature Portfolio guidelines for submitting code & software for further information.

Data

Policy information about availability of data

All manuscripts must include a data availability statement. This statement should provide the following information, where applicable:

- Accession codes, unique identifiers, or web links for publicly available datasets
- A description of any restrictions on data availability
- For clinical datasets or third party data, please ensure that the statement adheres to our policy

All data generated or analyzed during this study are included in this article and its Supplementary Information File. According to ethical permission and patient data-protection laws of Finland, single patient data cannot be made available.

Field-specific reporting

Please select the one below that is the best fit for your research. If you are not sure, read the appropriate sections before making your selection.

☒ Life sciences ☐ Behavioural & social sciences ☐ Ecological, evolutionary & environmental sciences

For a reference copy of the document with all sections, see nature.com/documents/nr-reporting-summary-flat.pdf

Life sciences study design

All studies must disclose on these points even when the disclosure is negative.

Sample size	In this real-life 12-year follow-up study the total cohort consisted of 259 patients having new-onset asthma that was diagnosed at adult age. Two patients were excluded because they were later found to have a previous diagnosis of asthma during childhood, leaving 257 patients in the original cohort. More than 94% of the patients diagnosed with novel asthma in the study site were recruited to the study. In 2001, the study population represented >38% of novel diagnoses of asthma made to adults in the whole geographical area.
Data exclusions	Exclusion criteria: Physical or mental inability to provide signed informed consent OR/AND Diagnosis of asthma below the age of 15 years.
Replication	Experimental studies were not carried out.
Randomization	No randomization was made.
Blinding	No blinding was made.

Reporting for specific materials, systems and methods

We require information from authors about some types of materials, experimental systems and methods used in many studies. Here, indicate whether each material, system or method listed is relevant to your study. If you are not sure if a list item applies to your research, read the appropriate section before selecting a response.

Materials & experimental systems

n/a	Involved in the study	
☒	☐	Antibodies
☒	☐	Eukaryotic cell lines
☒	☐	Palaeontology and archaeology
☒	☐	Animals and other organisms
☐	☒	Human research participants
☐	☒	Clinical data
☒	☐	Dual use research of concern

Methods

n/a	Involved in the study	
☒	☐	ChIP-seq
☒	☐	Flow cytometry
☒	☐	MRI-based neuroimaging

Human research participants

Policy information about studies involving human research participants

Population characteristics	The research population consists of adult patients (age >15) with new-onset asthma diagnosed by a respiratory specialist based on typical symptoms and was confirmed by objective lung function measurements. Smokers and patients with concomitant COPD or other comorbidities were not excluded.
Recruitment	In the original study cohort patients (n=257) were recruited between October 6th 1999 and April 17th 2002 from the diagnostic visit in Seinäjoki Central Hospital respiratory department (phase I). After the diagnosis was confirmed and the medication started the patients were treated and monitored by their personal physicians mostly in primary health care according to the Finnish National Asthma Programme. Phase I was originally designed as a registry serving as an asthma-related data exchange platform between primary and specialised care, as well as an asthma-related research registry. Phase I was part of hospital development projects (institutional permission TU 1114). No interventions outside normal clinical practice were carried out. All participants in the original cohort gave written informed consent to be included in the registry.<br><br>After 12 years (mean 12.2, range 10.8-13.9) a total of 203 patients completed a follow-up visit in respiratory department (phase II). Asthma status, disease control, comorbidities and medication were evaluated using structured questionnaires (AQ20, Asthma Control Test ATC) and lung function was measured. The participants of the follow-up visit gave written informed consent to the study protocol approved by the Ethics committee of Tampere University Hospital, Tampere, Finland (R12122).
Ethics oversight	Study protocol has been approved by the Ethics committee of Tampere University Hospital, Tampere, Finland.

Note that full information on the approval of the study protocol must also be provided in the manuscript.

Clinical data

Policy information about clinical studies

All manuscripts should comply with the ICMJE guidelines for publication of clinical research and a completed CONSORT checklist must be included with all submissions.

Clinical trial registration	NCT02733016
Study protocol	The details of the SAAS study protocol with specific diagnostic criteria has been published separately previously: Kankaanranta, H., Ilmarinen, P., Kankaanranta, T. & Tuomisto, L. E. Seinajoki Adult Asthma Study (SAAS): a protocol for a 12-year real-life follow-up study of new-onset asthma diagnosed at adult age and treated in primary and specialised care. NPJ Prim. Care Respir. Med. 25:15042 (2015).
Data collection	The study cohort patients (n=257) were recruited between between October 1999 and April 2002 from the diagnostic visit in Seinäjoki Central Hospital respiratory department and after 12 years a total of 203 patients completed a follow-up visit in respiratory department (between 10 December 2012 and 31 October 2013). In addition to the data gathered at these visits,<br>data on asthma follow-up visits, exacerbations, hospitalisations, possible occupationally induced asthma and prescribed asthma medication were collected from hospital clinics, primary health care, occupational health care and private practices for the whole 12-year follow-up period. Medication information was collected from medical records and from the Finnish Social Insurance Institution from the whole 12-year follow-up period.
Outcomes	The main aim of this study was to describe how smoking and pack-years were documented during scheduled asthma contacts in primary health care during 12-year follow-up, and if differences exist between contacts with GP, nurse, or both. The second aim was to evaluate how often patients were advised in smoking cessation and to assess how smoking status affected their asthma control and health care use.<br>To assess these outcomes all asthma-related health care contacts of the 203 patients during the 12-year follow-up period were explored. Of those we included planned primary health care (public health care centres and occupational health care) asthma follow-up contacts of 152 patients, the total number of contacts being 603. The excluded 51 patients didn't have any scheduled asthma contacts in primary health care. The data of 152 patients and the data gathered from their planned asthma contacts in primary health care was evaluated.
