# Supplementary material for: Documentation of smoking in scheduled asthma contacts in primary health care: a 12-year follow-up study
Source: NPJ Prim Care Respir Med. 2022 Oct 21;32:44. doi: 10.1038/s41533-022-00309-4 (PMC9587006; doi:10.1038/s41533-022-00309-4)
Supplement: Supplementary file 2 — Supplementary Material [file 41533_2022_309_MOESM2_ESM.pdf]

Supplementary information

Documentation of smoking in scheduled asthma contacts in primary health care: a 12-year follow-up study

Jaana Takala, MD, GP[1,2,3*], Iida Vähätalo, M.Sc.Pharm[2,3], Leena E. Tuomisto, MD, PhD[2,3], Onni Niemelä, MD, PhD [4,5], Pinja Ilmarinen, PhD[2,3], Hannu Kankaanranta, MD, PhD[2,3,6]

[1]Department of Internal Medicine, Seinäjoki Central Hospital, Seinäjoki, Finland

[2]Department of Respiratory Medicine, Seinäjoki Central Hospital, Seinäjoki, Finland

[3]Tampere University Respiratory Research Group, Faculty of Medicine and Health Technology, Tampere University, Tampere, Finland

[4]Department of Laboratory Medicine, Seinäjoki Central Hospital, Seinäjoki, Finland

[5]Tampere University, Tampere, Finland

[6]Krefting Research Center, Department of Internal Medicine and Clinical Nutrition, Institute of Medicine, University of Gothenburg, Gothenburg, Sweden

Corresponding author: Jaana Takala, MD, GP
Department of Respiratory Medicine
Seinäjoki Central Hospital
Hanneksenrinne 7
FIN-60220 Seinäjoki, FINLAND
e-mail: jaana.takala@fimnet.fi

Supplementary Table 1. Exclusion and inclusion criteria used in SAAS-study.

Inclusion criteria<br>• A diagnosis of new-onset asthma made by a respiratory specialist<br>• Diagnosis confirmed by at least one of the following objective lung function measurements<br>▪ FEV_1 reversibility in spirometry of at least 15 % and 200 ml<br>▪ Diurnal variability (⩾20%) or repeated reversibility (⩾15%/60 l/min) in PEF follow-up<br>▪ A significant decrease in FEV_1 (15%) or PEF (20%) in response to exercise or allergen<br>▪ A significant reversibility in FEV_1 (at least 15% and 200 ml) or significant mean PEF change in response to a trial with oral or inhaled glucocorticoids<br>• Symptoms of asthma<br>• Age ≥ 15 years
Exclusion criteria<br>• Physical or mental inability to provide signed informed consent<br>• Diagnosis of asthma below the age of 15 years

Reference: Kankaanranta, H., et al. Seinäjoki Adult Asthma Study (SAAS): a protocol for a 12-year real-life follow-up study of new-onset asthma diagnosed at adult age and treated in primary and specialised care. *NPJ Prim. Care Respir. Med.* **25:**15042 (2015).

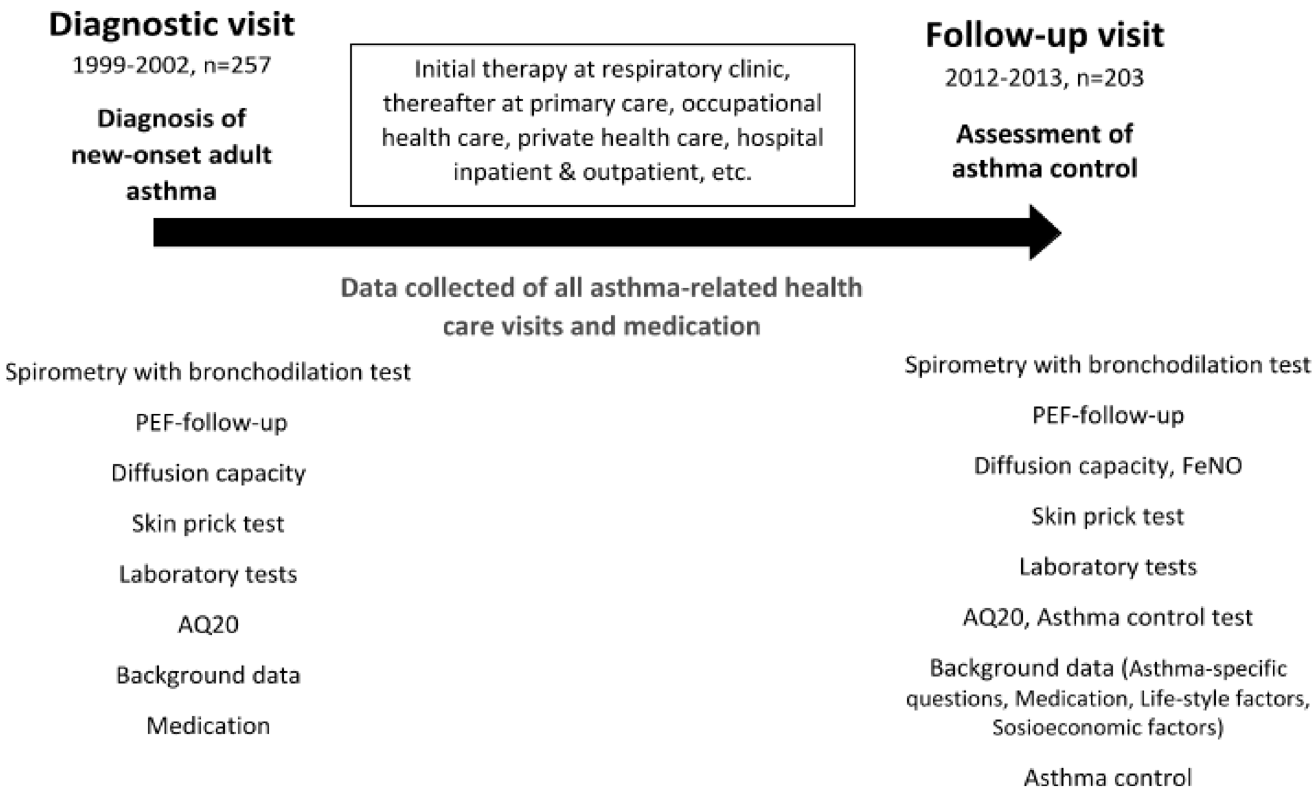

Supplementary Figure 1. Flowchart of Seinäjoki Adult Asthma Study.

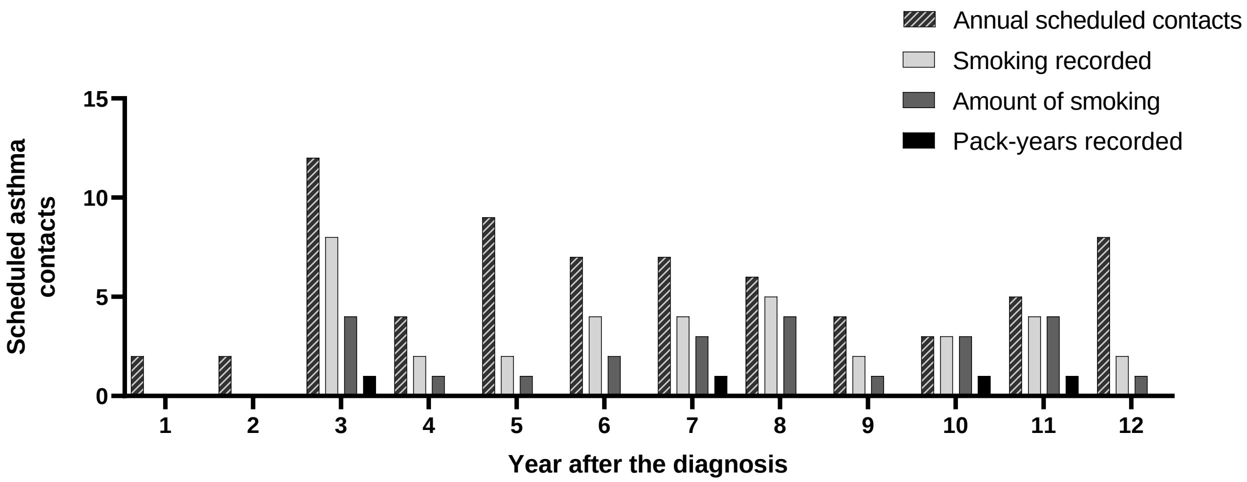

Supplementary Figure 2. Smoking data recording during 12-year follow-up for patients being current smokers at study baseline. The total number of all scheduled asthma contacts of current smokers (n=22) in primary health care was 69.

Supplementary Table 2. Additional characteristics of the study groups according to the baseline smoking status at 12-year follow-up visit.

	Never smoker n=79	Ex- or current smoker n=73	P-value
**Atopic n (%) [a]**	28 (38.9)	23 (35.4)	0.725
**Rhinitis n (%)**	57 (72.2)	52 (71.2)	>0.999
**Severe asthma n (%) [b]**	3 (3.8)	6 (8.2)	0.313
**Asthma therapy steps (GINA 2019) n (%) [c]**			
**- step 1-2**	13 (16.5)	15 (20.5)	
**- step 3**	23 (29.1)	16 (21.9)	0.764
**- step 4**	16 (20.3)	16 (21.9)	
**- step 5**	16 (20.3)	14 (19.2)	
**AQ20 score**	4 (2-7)	4 (2-7)	0.645
**Blood eosinophils (x10^9/l)**	0.15 (0.09-0.28)	0.16 (0.10-0.27)	0.915
**Blood neutrophils (x10^9/l)**	3.7 (2.7-4.8)	3.7 (2.9-4.7)	0.929
**Total IgE (kU/l)**	61.0 (23.0-166.0)	58.0 (23.5-153.5)	0.744
**FeNO (ppb)**	12.0 (6.8-19.3)	10.0 (5.0-17.3)	0.118
**Pre-BD FVC (%)**	100.8 (15.1)	96.3 (13.5)	0.057
**Post-BD FVC (%)**	101.3 (15.3)	98.6 (14.2)	0.264
**Post-BD FEV_1 (%)**	94.9 (15.0)	86.2 (17.6)	**0.001**
**Pre-BD FEV_1/FVC**	0.75 (0.68-0.80)	0.72 (0.64-0.76)	**0.008**
**FEV_1 reversibility (ml)**	80 (20-140)	100 (25-185)	0.117
**FEV_1 reversibility (%)**	2.76 (0.63-5.86)	4.05 (1.08-6.82)	0.265
**Number of comorbidities (COPD included)**	1 (0-2)	1 (0-3)	0.145
**Metabolic syndrome n (%)**	8 (10.1)	5 (6.9)	0.569
**Diabetes n (%)**	9 (11.4)	10 (13.7)	0.807
**Hypertension n (%)**	25 (31.6)	24 (32.9)	>0.999
**Ischemic heart disease n (%)**	6 (7.6)	12 (16.4)	0.131
**Any psychiatric disease n (%)**	9 (11.4)	11 (15.1)	0.632
**Thyroid disease n (%)**	6 (7.6)	8 (11.0)	0.579
**Painful condition n (%)**	10 (12.7)	7 (9.6)	0.613
**Treated dyspepsia n (%)**	9 (11.4)	4 (5.5)	0.251
**Purchased antibiotic courses during the follow-up**	7 (4-14)	8 (4-14)	0.779
**≥2oral corticosteroid course for asthma during 2-year before follow-up n (%)**	12 (15.6)	13 (18.1)	0.827
**≥1 hospitalization due to any respiratory related reason n (%)**	20 (25.3)	19 (26.0)	>0.999
**≥1 hospitalization due to asthma n (%)**	17 (21.5)	16 (21.9)	>0.999
**All asthma-related health care visits during 12-year follow-up**	15 (9-23)	17 (12-25)	0.271
**All scheduled asthma contacts after 2002**	4 (2-8)	4 (2-7)	0.545
**All scheduled asthma contacts**	7 (5-11)	7 (4-11)	0.808
**Unscheduled contacts**	4 (1-10)	4 (1-11)	0.315
**In working life n (%)**	36 (45.6)	30 (41.1)	0.625
**Length of education ≥ 12 years n (%)**	25 (31.6)	15 (20.5)	0.142
**Income (€)**	2000 (1200-2593)	2000 (1500-2500)	0.601

If not otherwise mentioned shown are mean (SD) or median (25th -75th) percentiles. AQ20 = Airway questionnaire, FeNO = fraction of NO in exhaled air, BD = bronchodilator, FVC = forced vital capacity, FEV_1 = forced expiratory volume in 1 s. [a]At least one positive skin prick test of common allergens. [b]Assessment of severe asthma was performed according to the ERS/ATS severe asthma guideline 2014.[c]Classification of asthma therapy steps was made based on daily medication regimen according to the GINA 2019 guideline. The GINA step could not be determined in 23 patients because of the lack of medication purchased.

Supplementary Table 3. Additional characteristics of ex-smokers and current smokers at follow-up visit 2013.

	Ex smoker<br>n= 57	Current smoker<br>n=19	P-value
**Rhinitis n (%)**	43 (75.4)	11 (57.9)	0.157
**Atopic n (%)** [a]	18 (35.3)	6 (35.3)	>0.999
**Blood eosinophils (x10^9/l)**	0.18 (0.11-0.29)	0.13 (0.09-0.24)	0.140
**Blood neutrophils (x10^9/l)**	3.60 (2.85-4.50)	4.20 (3.30-5.80)	0.055
**Total IgE (kU/l)**	60.0 (24.0-150.5)	43.0 (21.0-204.0)	0.679
**Asthma therapy steps (GINA 2019) n (%)** [b]<br>**- step 1-2**<br>**- step 3**<br>**- step 4**<br>**- step 5**	 11 (19.3)<br>13 (22.8)<br>13 (22.8)<br>10 (17.5)	 4 (21.1)<br>4 (21.1)<br>3 (15.8)<br>4 (21.1)	0.928
**AQ20 score**	5 (2-7)	4 (1-7)	0.759
**Self-reported daily ICS in use n (%)**	42 (73.7)	15 (78.9)	0.766
**Average daily prescribed ICS dose among 12-years (µg budesonide equivalents)**	810 (687-1005)	937 (637-1223)	0.410
**Daily LABA in use n (%)**	28 (49.1)	10 (52.6)	>0.999
**Add-on-drug in daily use n (%)**	30 (52.6)	12 (63.2)	0.595
**SABA puffs/week**	2.0 (0.95-4.37)	2.6 (1.09-5.03)	0.487
**Number of asthma or/and allergy medication in use**	2 (2-3)	2 (2-3)	0.710
**All asthma-related health care visits during 12-year follow-up**	17 (11-25)	18 (15-24)	0.272
**Scheduled asthma contacts after 2002**	4 (2-7)	4 (2-6)	0.966
**All scheduled asthma contacts**	7 (4-11)	7 (5-10)	0.895
**Pre-BD FVC (%)**	96 (14.6)	98 (10.6)	0.723
**Pre-BD FEV_1 (%)**	81 (19.1)	88 (11.7)	0.189
**Post-BD FVC (%)**	98 (15.0)	101 (12.3)	0.479
**Post-BD FEV_1 (%)**	85 (19.3)	91 (10.5)	0.228
**Pre-BD FEV_1/FVC**	0.72 (0.63-0.76)	0.75 (0.70-0.79)	0.116
**Post-BD FEV_1/FVC**	0.73 (0.66-0.79)	0.76 (0.70-0.80)	0.322
**FEV_1 reversibility (ml)**	90 (15-195)	130 (30-160)	0.801
**FEV_1 reversibility (%)**	3.6 (1.1-6.9)	4.1 (0.9-6.9)	0.848
**Annual change in lung function from $Max_{0-2,5}$ to follow-up** [c]<br>- **FEV_1 (ml/y)**<br>- **FEV_1 %/y**	 -49.1 (-68.7 to -24.7)<br>-0.60 (-1.18 to -0.04)	 -54.1 (-61.8 to -26.1)<br>-0.62 (-1.16 to -0.26)	 0.871<br>0.924
**Number of comorbidities**	1 (0-3)	1 (0-3)	0.715
**Metabolic syndrome n (%)**	5 (8.8)	0	0.329
**Diabetes n (%)**	9 (15.8)	1 (5.3)	0.436
**Hypertension n (%)**	20 (35.1)	4 (21.1)	0.393
**Ischemic heart disease n (%)**	9 (15.8)	3 (15.8)	>0.999
**Any psychiatric disease n (%)**	6 (10.5)	5 (26.3)	0.130
**Painful condition n (%)**	7 (12.3)	1 (5.3)	0.671
**Treated dyspepsia n (%)**	3 (5.3)	1 (5.3)	>0.999
**Income (€)**	2150 (1500-2600)	1915 (1400-2425)	0.482
**Marital status**<br>**-divorced/widow/no relationship n (%)**	9 (15.8)	6 (31.6)	0.183

If not otherwise mentioned shown are mean (SD) or median (25th -75th percentiles). AQ20 = Airway questionnaire, ICS = inhaled corticosteroid, LABA = long-acting β_2-agonist, Add-on drug = long-acting β_2-agonist, leukotriene receptor antagonist, theophylline and/or tiotropium in daily use, SABA = short-acting β_2-agonist, BD = bronchodilator, FVC = forced vital capacity, FEV_1 = forced expiratory volume in 1 s. [a]At least one positive skin prick test of common allergens. [b]Classification of asthma therapy steps was made based on daily medication regimen according to the GINA 2019 guideline. The GINA step could not be determined in 14 patients because of the lack of medication purchased. [c]Annual change in FEV_1 during 12 years of follow-up (ΔFEV_1 from point of maximal lung function within 2.5 years after start of therapy to the 12-year follow-up visit).
